# Extracellular Matrix Induces Periodontal Ligament Reconstruction In Vivo

**DOI:** 10.3390/ijms20133277

**Published:** 2019-07-03

**Authors:** Naoko Nakamura, Ai Ito, Tsuyoshi Kimura, Akio Kishida

**Affiliations:** 1 College of Systems Engineering and Science, Shibaura Institute of Technology, 307 Fukasaku, Minuma-ku, Saitama-shi, Saitama 337-8570, Japan; 2 Institute of Biomaterials and Bioengineering, Tokyo Medical and Dental University, 2-3-10 Kanda-Surugadai, Chiyoda-ku, Tokyo 101-0062, Japan

**Keywords:** decellularization, periodontal ligament, reconstruction, extracellular matrix

## Abstract

One of the problems in dental implant treatment is the lack of periodontal ligament (PDL), which supports teeth, prevents infection, and transduces sensations such as chewiness. The objective of the present study was to develop a decellularized PDL for supporting an artificial tooth. To this end, we prepared mouse decellularized mandible bone with a PDL matrix by high hydrostatic pressure and DNase and detergent treatments and evaluated its reconstruction in vivo. After tooth extraction, the decellularized mandible bone with PDL matrix was implanted under the subrenal capsule in rat and observed that host cells migrated into the matrix and oriented along the PDL collagen fibers. The extracted decellularized tooth and de- and re-calcified teeth, which was used as an artificial tooth model, were re-inserted into the decellularized mandible bone and implanted under the subrenal capsule in rat. The reconstructed PDL matrix for the extracted decellularized tooth resembled the decellularized mandible bone without tooth extraction. This demonstrates that decellularized PDL matrix can reconstruct PDL tissue by controlling host cell migration, which could serve as a novel periodontal treatment approach.

## 1. Introduction

Periodontal ligament (PDL) connecting tooth and alveolar bone has several functions including preventing bacterial infection, shock absorption, sensing load on the teeth, and moving and aligning teeth. Periodontitis causes damage to tooth-supporting tissues such as PDL and alveolar bone, resulting in tooth loss. Dental implants are a general treatment for tooth loss for which several materials including Ti and Zr are used. Implants are directly inserted into and anchored in alveolar bone. To promote osseointegration, various types of modification of the implant surface have been attempted. Recent studies have also investigated the regeneration of PDL using stem cells. Stem cell sheets were shown to regenerate PDL in vivo [1,2], and periodontal stem cell sheets enhanced PDL regeneration around Ti dental implants [3].

Decellularized tissue, which is an extracellular matrix (ECM), is a candidate material for promoting tissue regeneration. Many techniques for decellularization have been developed and the appropriate method is selected based on the tissue of interest [4,5,6,7,8,9]. We previously reported that high hydrostatic pressure (HHP) decellularization, in which cells are ruptured by HHP and cell components are washed out, can effectively decellularize tissues such as blood vessel [10,11,12,13], cornea [14,15], and bone and bone marrow [16,17] while preserving the tissue structure. Additionally, HHP-decellularized tissues showed good biocompatibility and regenerative capacity. We also showed that bone was effectively regenerated in situ by matrix induction after subcutaneous implantation of HHP-decellularized bone.

Based on the above findings, we speculated that decellularized mandible bone consisting of PDL matrix and alveolar bone could serve as a tooth-supporting tissue. We prepared a decellularized mandible bone with PDL by HHP decellularization and investigated the re-cellularization of the PDL with the subrenal capsule assay in order to determine whether the ECM of the PDL can reconstruct the PDL matrix in the absence of PDL cells. Given the difficulty of detaching the very thin PDL from both teeth and bone, we prepared decellularized PDL on alveolar bone and evaluated the potential for PDL reconstruction. Although our final goal is to develop a material for PDL reconstruction, implanting a unit consisting of PDL and mandible bone is another promising strategy since many patients do not have a sufficient amount of alveolar bone and require a treatment that stimulates bone formation to support the dental implant; decellularized mandible bone with PDL matrix can be useful in such cases.

## 2. Results

### 2.1. Decellularization of Mandible Bone including Tooth

**Figure 1:**
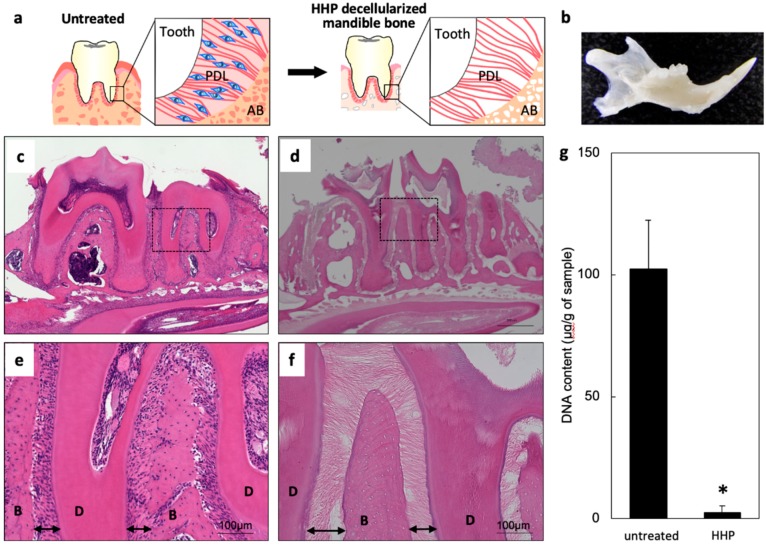
High hydrostatic pressure (HHP) decellularization of rat mandible bone. (**a**) Schematic illustration of the decellularization process. (**b**) Image of decellularized mandible bone. (**c**–**f**) Hematoxylin and eosin (HE)-stained sections of untreated (**c**,**e**) and HHP-decellularized (**d**,**f**) mandible bone. Arrows indicate the periodontal ligament (PDL) matrix area. B, alveolar bone; D, dentin. (**g**) Quantitative analysis of residual DNA (*n* = 4). * *p* < 0.05.

Mouse mandible bones including teeth were decellularized by HHP (Figure 1a,b). Many cells were observed in the untreated sample by hematoxylin and eosin (HE) staining (Figure 1c,e); however, there were no cells in the bone or PDL following decellularization (Figure 1d,f). Collagen fibers were clearly visible between the roots of teeth and alveolar bone, and this connection remained intact after decellularization. DNA was extracted from the untreated and decellularized samples and the concentration was measured with an ultraviolet–visible light spectrometer. The amount of residual DNA was lower for the decellularized as compared to the untreated sample (Figure 1g). These results demonstrate the successful HHP-induced decellularization of mandible bone with teeth.

### 2.2. Preparation of Mandible Bone with PDL Matrix

**Figure 2:**
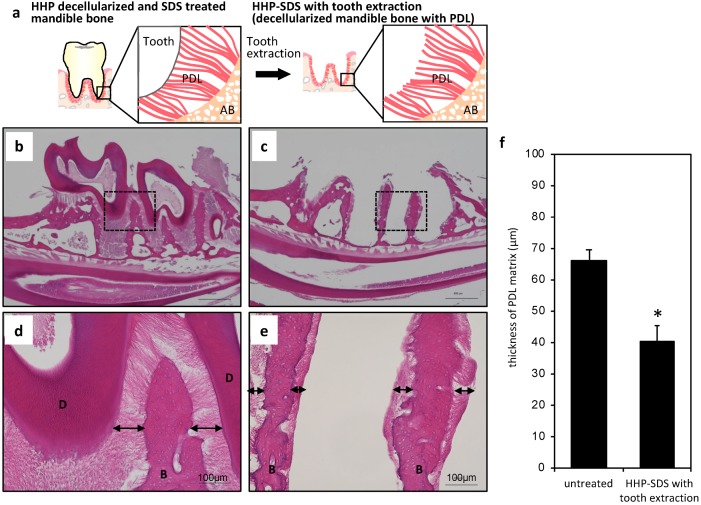
Sodium dodecyl sulfate (SDS) treatment and tooth extraction of decellularized mandible bone. (**a**) Schematic illustration of the tooth extraction process. (**b**–**e**) HE-stained images of HHP-decellularized (**b**,**d**) and SDS-treated (**c**,**e**) mandible bone with tooth extraction. Arrows indicate the PDL matrix area. B, alveolar bone; D, dentin. (**f**) Measurement of PDL matrix thickness (*n* = 5). * *p* < 0.05.

Teeth in the HHP-decellularized sample were extracted in order to prepare a decellularized mandible bone with PDL matrix (Figure 2a). This procedure was difficult because the teeth often broke. Additionally, even when the tooth was successfully extracted, most of the PDL matrix remained on the root of the extracted tooth and there was little left on the alveolar bone (~10 µm). We therefore treated the decellularized sample with sodium dodecyl sulfate (SDS), which can dissolve collagen fibers. After treatment with 0.3% SDS for 12 h, mandible bone tissue samples before and after tooth extraction were stained with HE. The PDL connecting the tooth and alveolar bone was maintained and there were no histological changes in the PDL before tooth extraction from the decellularized mandible bone treated with SDS (Figure 2b,d). After the treatment, the tooth was easily extracted and PDL was observed on the alveolar bone (Figure 2c,e). The thickness of the PDL on the alveolar bone in the untreated sample was about 71 ± 4 µm; after tooth extraction, this was reduced to 40 ± 4 µm due to division and shrinkage (Figure 2f). Thus, decellularized mandible bone with PDL matrix was obtained by HHP decellularization combined with SDS treatment.

### 2.3. In Vivo Re-cellularization of HHP-decellularized Mandible Bone

**Figure 3:**
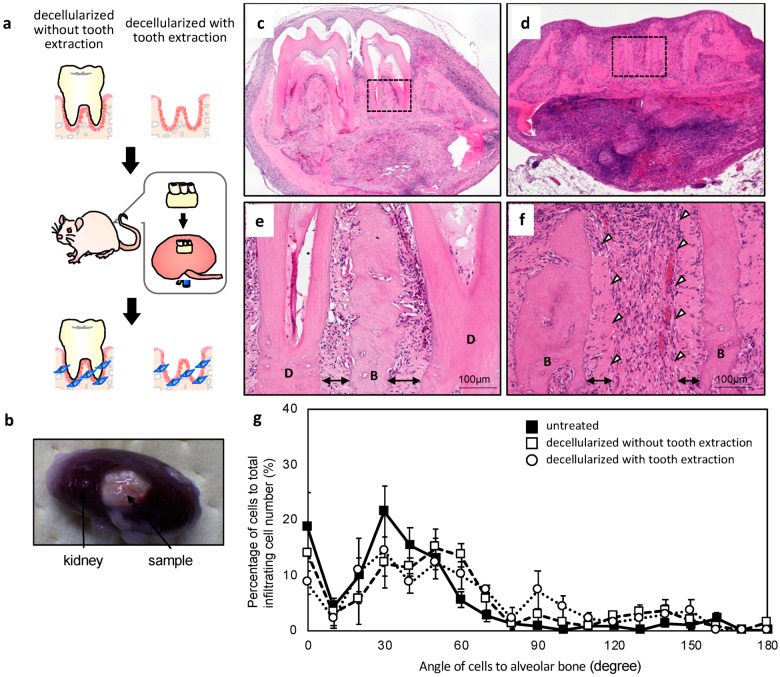
Implantation of decellularized mandible bone without and with tooth extraction. (**a**) Schematic illustration of the implantation process. (**b**) Implanted sample three weeks after the subrenal capsule assay. (**c**–**f**) HE-stained images of subrenal implantation of decellularized mandible bone without (**c**,**e**) and with (**d**,**f**) tooth extraction. Arrows indicate the PDL matrix area. White arrowheads show the surface of the PDL matrix. B, alveolar bone; D, dentin. (**g**) Angle of cells infiltrated into the PDL matrix relative to alveolar bone (*n* = 5).

We used the subrenal capsule assay to evaluate the re-cellularization of the HHP-decellularized mandible bone with and without tooth extraction (Figure 3a). Although a subcutaneous implantation model is typically used to evaluate the biocompatibility of biomaterials and their potential for tissue reconstruction, one study reported the regeneration of tooth using tooth germ and PDL reconstruction by subrenal implantation, which is generally used to test drug efficacy in xenografted tumors [18]. We therefore compared subcutaneous implantation with the subrenal capsule assay. Since significant neovascularization in the PDL matrix was observed in the latter, this assay was adopted in the present study. The implanted samples were harvested after three weeks and it was visually confirmed that many vessels were generated around the implanted samples (Figure 3b). Histological evaluation also showed red blood cells and neomicrovascular cross sections were observed in the collagen gel area and the decellularized sample (Figure 3c–f). Many nucleated cells were also observed in the both samples (Figure 3c–f). Without tooth extraction, the PDL connected the alveolar bone to teeth, and migrated cells were detected in the PDL matrix and showed typical orientation (Figure 3c,e). There is no evidence of inflammation. For the decellularized sample with tooth extraction, the space that was previously occupied by the tooth was filled with cells; the PDL was intact, and migrated cells were present in the PDL matrix (Figure 3d,f) that were oriented along the collagen fibers, similar to those in the PDL matrix of mandible bone without tooth extraction (Figure 3g). This suggests that cells migrated along the PDL matrix.

### 2.4. Preparation of an Artificial Tooth Model

**Figure 4:**
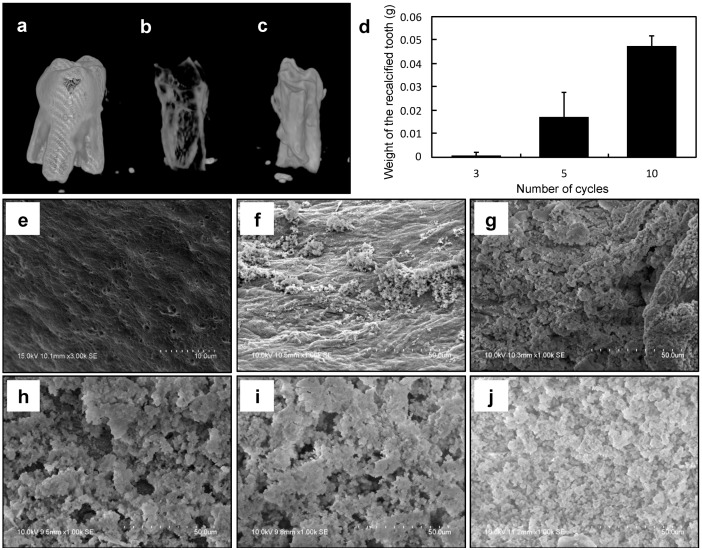
(**a**–**c**) Micro-computed tomography (μCT) images of extracted decellularized tooth (**a**), decalcified tooth (**b**), and re-calcified tooth (**c**). (**d**) Measurement of weight after alternate soaking. (**e**–**j**) Scanning electron microscopy (SEM) images of modified tooth surface of the decalcified sample (**e**) and after three (**f**), four (**g**), six (**h**), eight (**i**), and 10 (**j**) cycles.

We investigated whether a decellularized mandible bone with PDL matrix and a re-inserted artificial tooth could serve as artificial periodontal tissue. The extracted tooth was used as the artificial tooth since it fit well into the space left by the removed tooth. The artificial tooth was prepared by de-calcifying and then re-calcifying the extracted tooth by the alternate soaking method. The calcification of the extracted tooth was confirmed by micro-computed tomography (μCT), scanning electron microscopy (SEM), and by the change in weight. Before de-calcification, the shape of the crown and root of the extracted tooth were visible by μCT imaging (Figure 4a); however, it was difficult to discern the de-calcified tooth, especially since the crown had disappeared (Figure 4b). After re-calcification, the shape of the tooth was again apparent and the root was mostly preserved (Figure 4c). SEM analysis revealed more calcium phosphate particles on the re-calcified tooth root surface with an increasing number of alternate soaking cycles (Figure 4e–j); a similar trend was observed for the weight of the extracted tooth (Figure 4d).

### 2.5. In Vivo Re-cellularization of Decellularized Mandible Bone with Re-inserted Artificial Tooth

**Figure 5:**
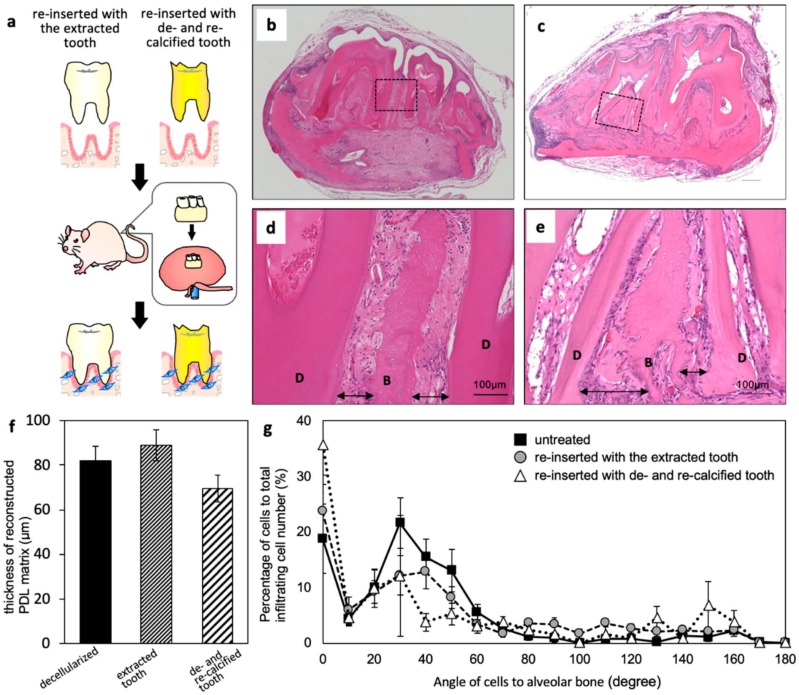
Implantation of decellularized mandible bone re-inserted with the extracted tooth or with de-/re-calcified tooth. (**a**) Schematic illustration of the implantation process. (**b**–**e**) HE-stained images of subrenal implantation of decellularized mandible bone re-inserted with the extracted (**b**,**d**), and with de-/re-calcified (**c**,**e**) tooth. Arrows show the PDL matrix area. B, alveolar bone; D, dentin. (**f**) Thickness of the reconstructed PDL matrix after the subrenal capsule assay (*n* = 3). (**g**) Angle of cells infiltrated into PDL matrix relative to alveolar bone (*n* = 5).

We investigated the re-cellularization of decellularized PDL with re-inserted artificial tooth with the subrenal capsule assay (Figure 5a). When the decellularized mandible bone with the re-inserted artificial tooth without de- and re-calcification was implanted as a control, we observed that cells had migrated into the PDL matrix, which is similar to the situation in decellularized mandible bone without tooth extraction (Figure 5b,d). We measured the thickness of the reconstructed PDL matrix in HE-stained sections and found that it was the same in decellularized mandible bone with the re-inserted tooth and without tooth extraction (Figure 5f). The migrated cells were aligned along the collagen fibers (Figure 5g). In the case of artificial tooth, cells were observed in the PDL, although there was no difference in PDL thickness; in fact, the PDL was somewhat thin (Figure 5c,e,f). In addition to the cells aligned along the collagen fibers of the PDL matrix, many cells were observed close to the surface of the artificial tooth and were randomly oriented (Figure 5g).

## 3. Discussion

PDL regeneration can improve the quality of life of dental implant patients by compensating for the loss of PDL function. Some studies have proposed a PDL cell sheet for PDL reconstruction; inserting these sheets between the native tooth and β-tricalcium phosphate granules induced the regeneration of PDL collagen fibers [1,2], while PDL reconstructed by tooth germ surrounding the dental implant root had the ability to move the tooth in response to a load [18,19]. In these methods, PDL was reconstructed using cells derived from or that can differentiate into PDL cells. In clinical practice, a treatment strategy has been established whereby a tooth is transplanted into a defect site and becomes engrafted when living PDL cells attach to its root. Thus, cells constituting the PDL are important for PDL regeneration.

Tissue regeneration in situ without using cells has also been attempted. In this study, we developed a tooth-supporting decellularized mandible bone with PDL and investigated PDL tissue regeneration by the decellularized mandible bone with PDL serving as a scaffold that can recruit the appropriate cells and induce their correct orientation within the PDL. The mandible bone was decellularized with the HHP method [16,17]. We confirmed cell removal from the PDL and the preservation of the PDL ECM. As the DNA contents in the HHP treated mandible bone was under 50 ng per mg ECM dry weight, of which the contents are defined by a previous report [6], our sample would be applied for in vivo assay. Decellularized mandible bone with PDL matrix was obtained by SDS treatment and tooth extraction. When we attempted to extract a tooth from the decellularized mandible without SDS treatment, the tooth broke, leaving behind its root; or else the PDL was removed along with the extracted tooth since it is strongly connected to both tooth and bone. SDS—is a strong detergent that can dissolve cells and ECM—weakened the connection between the tooth and PDL, however, SDS treatment conditions must be optimized for each tissue. Although SDS treatment allowed tooth extraction without root breakage, the PDL matrix on alveolar bone was dissolved by 1.0% SDS treatment for 24 h. The matrix remained intact by treatment with 0.3% SDS for 12 h, although the thickness was decreased under this condition. One reason for this is that a small amount of PDL matrix was removed along with the extracted tooth; another is that there was no tensile stress on collagen fibers constituting the PDL matrix after tooth extraction, resulting in their shrinkage due to a lack of connection with the tooth.

We used the subrenal capsule assay to test the PDL regeneration capacity of our model since it is a robust microvascular network and had abundant blood—both of which are essential for tissue regeneration—as observed with this assay. Numerous blood vessels and cells were visible in implanted samples consisting of decellularized mandible bone and collagen gel; most of cells were spindle-shaped. Decellularized mandible bones without and with tooth extraction exhibited cell infiltration into the PDL matrix and had an intact collagen fiber structure. This indicates that ECM-based decellularization can be maintained in vivo without degradation.

Native PDL cells in untreated PDL matrix were oriented at 20°–50° relative to the long axis of collagen fibers, with the peak of the distribution of orientation angles at 30°. Migrated cells in decellularized mandible bone without and with tooth extraction showed a similar orientation as untreated PDL cells, suggesting that this was controlled by decellularized PDL collagen fibers. In previous studies, host cells were shown to home to the appropriate location [20,21]. We also reported that specific cellular engraftment and tissue regeneration was induced using suitable three-dimensional ECM structures [17]. Cells that migrate into the decellularized PDL matrix may participate in PDL reconstruction.

There was no obvious inflammatory response at the implantation site of decellularized PDL and mandible bone, indicating that there was no immune rejection. We previously reported that strong acute inflammation did not occur when HHP-decellularized porcine tissue was implanted into rabbits, rats, and mice [10,11,12,13,14,15,16,17]. Thus, HHP-decellularized mouse mandible bone with PDL is tolerated by rat host cells.

We prepared artificially modified tooth instead of a dental implant since we needed a tooth that could fit into the space previously occupied by the extracted tooth. We first decalcified the extracted teeth in order to alter their properties; the teeth were then re-calcified by the alternate soaking method to mimic the apatite coating of dental implants. A comparison of decellularized mandible bone with the extracted/re-inserted tooth and without tooth extraction revealed that decellularized PDL matrix in both specimens were fully re-cellularized, with no significant difference in their thickness (Figure 3e and Figure 5d,f). Additionally, cells in both samples showed similar orientation. The orientation of collagen fibers connecting the tooth and alveolar bone was slightly perturbed; we assumed that this was due to re-insertion of the artificial tooth since the PDL matrix structure was maintained in the sample without re-insertion (Figure 3f). Nonetheless, the collagen fiber connection between tooth and alveolar bone was partly restored. As mentioned above, autologous tooth transplantation with native PDL cells is performed in clinical practice and leads to engraftment. Here we showed that decellularized PDL matrix can induce PDL tissue regeneration by host cells even if there are no PDL cells before implantation. However, it should be noted that reconstruction is possible only with the appropriate materials.

When we compared the artificially modified tooth with the tooth that was extracted and re-inserted without de-/re-calcification, we found that the latter showed better PDL histological reconstruction and collagen fiber structure. In contrast, in the case of the artificial tooth, some areas of the PDL—especially on the bone side—were not re-cellularized and the collagen fiber structure was not restored; most cells were located around the surface of artificial tooth and were not uniformly oriented. This was likely due to cell mobilization in response to the calcium coating; that is, excessive migration could prevent the alignment of cells within collagen fibers of the decellularized PDL matrix. In order to achieve PDL reconstruction, a material for generating an artificial tooth that inhibits local cellular mobilization should be used.

One limitation of this study is that we evaluated PDL reconstruction histologically but did not examine the connection of collagen fibers to the tooth surface, localization of nerve cells and response to load. We plan to investigate these aspects in a future study. Here we report the reconstruction of PDL matrix from ECM.

## 4. Materials and Methods

### 4.1. Preparation of Decellularized Mandible Bone

Mandible bones were harvested from 5-week-old male C57BL/6 mice (Sankyo Lab Service Corporation, Nagano, Japan) and washed with saline. The bone was decellularized by HHP as previously reported [16,17]. Briefly, the bone was pressurized at 1000 MPa and 10 °C for 10 min using a cold isostatic pressurization machine (Dr. CHEF; Kobe Steel, Kobe, Japan). Pressurization and decompression were performed at 490 MPa/min, and propylene glycol was used as the transmission fluid. The bone was incubated for 4 weeks at 37 °C in saline containing 0.2 mg/mL DNase I (Roche, Indianapolis, IN, USA), 0.5 M MgCl_2_, and antibiotics, and then treated with saline containing 80% ethanol and incubated for 3 days at 37 °C with shaking, followed by pure saline for 3 days at 37 °C.

### 4.2. Preparation of PDL Matrix on Decellularized Mandible Bone

The decellularized mandible bone was treated with detergent to remove the teeth that were maintaining the PDL matrix on the bone. After incubation in 0.3% SDS solution with moderate shaking for 12 h, teeth on the mandible bone were extracted using forceps.

### 4.3. Histology

Samples were fixed in 10% neutral buffered formalin at room temperature for 24 h and then immersed in 0.24 M EDTA solution at 37 °C for 24 h with shaking for decalcification. Tissue samples were dehydrated through a graded series of ethanol, immersed in xylene, and embedded in paraffin. The blocks were sectioned at a thickness of 4 μm, and sections were deparaffinized and stained with Mayer’s HE.

### 4.4. Quantification of Residual DNA

Specimens were freeze-dried, weighed, cut, and digested overnight at 55 °C with 50 mg/mL proteinase-K in 50 mM Tris-HCl, 25 mM EDTA-2Na, 100 mM NaCl, and 1% SDS. DNA was extracted with the phenol/chloroform method followed by ethanol precipitation. DNA concentration was determined by measuring the absorbance at 260 nm.

### 4.5. Subrenal Capsule Assay

Animal experiments were approved by the Animal Care and Ethics Committee of Tokyo Medical and Dental University (approval nos. 0150268C: 1 April 2014, and 0160336C: 27 July 2015). 

Three molar parts of the decellularized mouse mandible were trimmed (5 × 4 × 2 mm) and embedded in 100 μL of collagen gel (KOKEN Co., Tokyo, Japan) according to the manufacturer’s instructions so that they could be easily inserted under the kidney capsule. Under deep anesthesia, the kidney of a female rat (150 g) was exposed; a 5- to 10-mm incision was made to the kidney capsule with a scalpel to create a pocket into which the samples were inserted. The samples were removed on day 21 post transplantation for analysis.

### 4.6. Surface Modification of Extracted Teeth

For decalcification, extracted teeth were immersed in 10% EDTA (pH 7.4) for 1 day at room temperature. For re-calcification, we used the alternate soaking method [22]. Briefly, extracted teeth were soaked in 200 mM CaCl_2_ solution at 37 °C for 1 min, rinsed with ultra-pure water at 37 °C for 1 min, soaked in 120 mM Na_2_HPO_4_ at 37 °C for 1 min, and rinsed with ultra-pure water at 37 °C for 1 min. These four steps were repeated 10 times.

### 4.7. SEM and Energy Dispersive X-ray Spectrometry

Samples were fixed at room temperature for 24 h in 2.5% glutaraldehyde in phosphate-buffered saline, dehydrated through a graded series of ethanol, immersed overnight in tert-butyl alcohol, and freeze-dried. The dried samples were sputter-coated with gold and observed by SEM using a model S-3400N microscope (Hitachi High-Technologies Corporation, Tokyo, Japan).

### 4.8. μCT

The specimens were imaged by X-ray (100 kV and 30 μA) using an inspeXio SMX-90CT scanner (Shimadzu, Kyoto, Japan).

### 4.9. Quantitative Analysis of Cell Orientation

The orientation of cells that migrated into the PDL matrix was examined in HE-stained images. The angle at which the long axis of blue-stained elliptical cell nuclei intersected with alveolar bone was measured.

### 4.10. Statistical Analysis

Results are expressed as mean ± standard deviation. Statistical significance was evaluated with the Student’s *t* test. *p* < 0.05 was considered statistically significant.

## 5. Conclusions

Decellularized mandible bone with PDL matrix was prepared that retained the collagen fiber structure; these fibers guided the migration and arrangement of host cells in vivo such that the cells were oriented in the same manner as the original PDL cells. The ECM derived from decellularized tissue was able to reconstruct the PDL tissue even in the absence of PDL cells. These results represent a significant advance in orthodontic treatment.
